# Natural polymerase fusion as an initiation regulator?

**DOI:** 10.18632/oncotarget.9565

**Published:** 2016-05-23

**Authors:** Guoliang Lu, Peng Gong

**Affiliations:** Key Laboratory of Special Pathogens and Biosafety, Wuhan Institute of Virology, Chinese Academy of Sciences, Wuhan, Hubei, China

**Keywords:** flavivirus, NS5, RNA-dependent RNA polymerase

Viral RNA-dependent RNA polymerases (RdRPs) are a unique class of enzymes that play the central role in replicating the genome of RNA viruses. The polymerase core of RdRP structurally resembles an encircled human right hand with palm, fingers, and palm domains surrounding the active site, with the unique fingers-thumb interactions to make the encirclement. Seven RdRP catalytic motifs have been identified based on sequence and/or structural homology, and the spatial organization of these motifs around the active site are highly analogous and key catalytic residues within the motifs are highly conserved [1, 2], making RdRPs overall the most conserved enzymes encoded by all RNA viruses. On the other hand, RdRPs are also diverse in several aspects including the organization of the polypeptide(s) harboring the polymerase activity. A typical polymerase core ofRdRP has a size about 50-70 kD. RdRPs such as poliovirus (PV) 3D^pol^ and hepatitis C virus (HCV) NS5B represent those not containing additional domains beyond the core. On the other extreme, RdRPs from negative­ strand RNA viruses have quite complicated architectures, with influenza virus three-subunit PA-PB1-PB2 replicase complex possessing both polymerase and endonuclease activities and the 250 kD vesicular stomatitis virus (VSV) L protein including at least three enzyme modules. The ≈ 105 kD flavivirus NS5 proteins fall between the two extremes with a ≈ 30 kD methyltransferase (MTase) naturally fused to theN-terminus of the RdRP through a short 10-residue linker, thus being an ideal system to study how the polymerase function is cis-regulated by its fusion partners.

Recently, we solved the full-length Japanese encephalitis virus (JEV) NS5 crystal structure, providing the first high resolution snapshot of MTase-RdRP interactions in flavivirus NS5 [3]. In such a structure, the MTase interacts with the fingers domain of RdRP, and the key residues within the intra-molecular MTase-RdRP interface are highly conserved in the Flavivirus genus. Compared to the MTase-less NS5 structures, the ring and pinky subdomains within the fingers domain become fully ordered and adopts classical folds observed in RdRP catalytic complexes [4], readying for their function in template RNA binding and NIP binding, respectively. The conservation of the MTase-RdRP interactions and the consequence brought by the interactions to shape the RdRP strongly imply the regulatory roles of MTase in RdRP function. To better understand the MTase-RdRP crosstalk, we designed mutations to perturb the interface in JEV full-length virus and replicon systems and found that virus replication was significantly inhibited, and further tests in dengue virus serotype 2 (DENV-2) confirmed the importance and conservative roles of these MTase-RdRP interactions [5]. We next introduced interface mutations and N-terminal deletions in JEV NS5 and used in vitro polymerase assays to dissect how the MTase-RdRP interactions modulate the process of RNA synthesis [6]. As with other RdRPs utilizing the de novo initiation mechanism, the flavivirus NS5 needs to undergo an unstable initiation phase before completing a transition to the processive elongation phase, presumably when the template-product RNA duplex reaches a minimum length of 7-8 base pairs [7] (Figure 1). We utilized a primer-dependent assay and a dinucleotide-driven de novo assay to assess unstable initiation, and performed a high-salt chase assay to probe the properties of a more stable polymerase complex that is likely in the late stages of the transition process toward the elongation phase. The mutations within the MTase-RdRP interface showed consistent up- or down- regulation of RNA synthesis in both initiation assays, indicating the important roles of the MTase-RdRP interactions in unstable initiation complexes (ICs). Interestingly, the interface mutations that exhibited the most prominent effect in the initiation assays did not have the similar impact on the relatively stable complex. Among the four NS5 mutants tested, two showed elongation rates comparable to that of the wild type enzyme, and the other two rather had effects opposite to what observed in the initiation assays.

**Figure 1:**
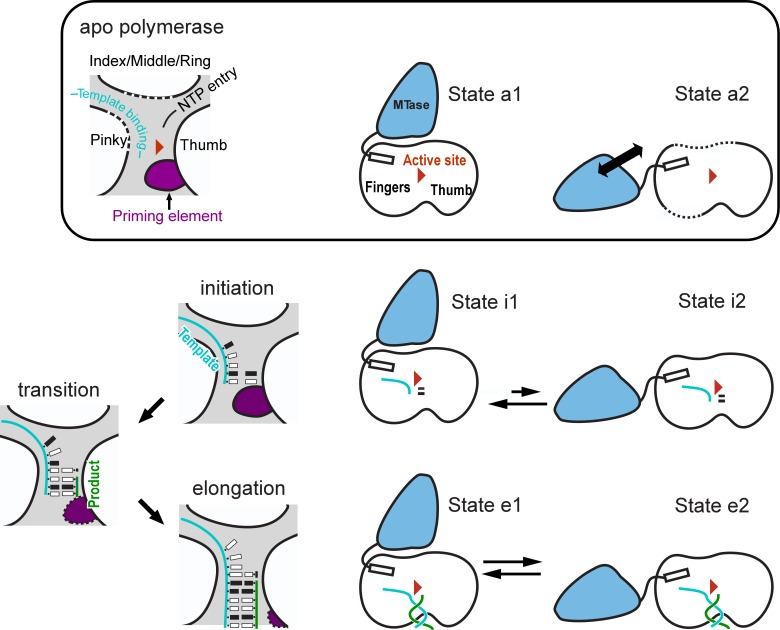
Different conformational states of flavivirus NS5 and their roles in polymerase catalysis Top) apo NS5 may exist in two general states: a State al with the polymerase fingers domain properly folded; a State a2 with the pinky/ring finger subdomains disordered to various extent. Bottom left) A scheme describing the transition from initiation to elongation in RNA synthesis. The priming element plays a critical role in the early stages, and may gradually withdraw from the active site to accommodate the growth of the template product RNA duplex during the transition process. Bottom right) A hypothesis: the polymerase activity of non-ECs may be susceptible to NS5 conformational changes, while the polymerase activity of a *bona fide* EC may not be influenced by the global conformation of NS5.

The assays we utilized in this study likely allow us to probe polymerase initiation and the transition process up to the late stages, but clearly not the *bona fide* elongation process, as the polymerization rate constants we measured were not large enough to match the typical 10-100 nt/sec constants observed in other polymerase elongation complexes (ECs). Our tentative conclusion is that MTase regulates RdRP catalysis mainly during the early stages of synthesis. The regulation differences observed between the unstable complex and relatively stable complex may reflect the change in the equilibrium among conformational states of NS5 (Figure 1). We propose that MTase may have minimum regulatory effect in a *bona fide* EC that have established optimum polymerase-nucleic acid interactions for processive RNA synthesis. Further clarification of these uncertain aspects likely requires solving high-resolution structures of full­ length NS5 catalytic complex, both at the initiation and the elongation stages of RNA synthesis.

## References

[1] Wu J (2015). International journal of molecular sciences.

[2] Cerny J (2014). PloS one.

[3] Lu G, Gong P (2013). PLoS pathogens.

[4] Gong P (2010). Proceedings of the National Academy of Sciences of the United States of America.

[5] Li XD (2014). PLoS neglected tropical diseases.

[6] Wu J (2015). Journal ofviro1ogy.

[7] Malet H (2008). Antiviral research.

